# An Offline Digital-Twin-Assisted Decision-Support Framework for Dynamic RO Under Kuwait Solar-Availability Conditions

**DOI:** 10.3390/membranes16090281

**Published:** 2026-08-23

**Authors:** Fajer M. Alelaj, Mohammed A. Bou-Rabee, Mustafa Fadel, Shafqat Aziz, Adil Aslam Mir, Abdulrahman Alharbi, Hussain Al-Sairfi

**Affiliations:** 1 Department of Water Research Center, Kuwait Institute for Scientific Research (KISR), Kuwait City 13109, Kuwait; 2 Department of Electrical Engineering, College of Technical Studies, Public Authority for Applied Education and Training (PAAET), Kuwait City 13092, Kuwait; 3 College of Engineering, Mustansiriyah University, Baghdad 10052, Iraq; mustafa_1988abbas@uomustansiriyah.edu.iq; 4 E-Commerce Project Designer, Plymouth Hope, Plymouth PL4 8AA, UK; 5 Department of Computer Engineering, Ankara Yıldırım Beyazıt University, Ankara 06050, Turkey; 6 Department of Electrical Engineering, Jubail Industrial College, Jubail 31961, Saudi Arabia

**Keywords:** offline digital twin, dynamic pressure operation, permeate flow rate, Kuwait, machine learning, photovoltaic desalination, reverse osmosis, specific energy consumption

## Abstract

Reverse osmosis (RO) desalination is a major technology for freshwater production in arid regions, but its energy demand becomes more challenging when the system is supplied by variable renewable energy. This study presents an offline digital-twin-assisted decision-support framework for dynamic RO under Kuwait solar-availability conditions. Within this framework, the predictive models are driven primarily by the dynamic RO process variables, while NASA Prediction Of Worldwide Energy Resources (POWER) data provide the Kuwait solar-availability context, and the PV power margin serves as a scenario-level energy indicator. The purpose is to predict instantaneous permeate flow rate, estimate specific energy consumption, and identify energy-efficient operating conditions using machine learning. Kuwait City was used as the solar case-study location. Hourly solar and meteorological data were obtained from NASA POWER, while dynamic RO membrane data were obtained from the open experimental wave desalination dataset published by the National Renewable Energy Laboratory (NREL) through Data.gov and the Marine and Hydrokinetic Data Repository. The RO dataset includes steady-state, ramp, sinusoidal, and Wave Energy Converter SIMulator (WEC-Sim) pressure/flow experiments. The process-flow image used in the system description was also taken from the same NREL dataset and is cited in the figure caption. The raw RO files were cleaned, harmonized, and transformed into a process-informed modeling dataset. Derived features included pressure rate, recovery ratio, salt rejection, estimated pump power, specific energy consumption (SEC), PV power margin, and rolling pressure/flow features. Three supervised regression models were tested: Gradient Boosting, Random Forest, and XGBoost. A representative subset of 60,000 records was used to preserve the main experimental conditions while reducing redundancy in the densely sampled sequential data. Results show that permeate flow rate can be predicted with high accuracy using Gradient Boosting (R^2^ = 0.981; RMSE = 0.161 L/min). The moderate energy prediction performance yielded an R^2^ of 0.654 and RMSE of 7.570 kWh/m^3^ for Random Forest. The accuracy of permeate conductivity predictions was lower (R^2^ = 0.257; RMSE = 245.44 µS/cm) because membrane and feed characterizing parameters should be included for an adequate water quality control. The proposed approach is best suited as an offline decision-support framework for dynamic RO process analysis.

## 1. Introduction

Water scarcity is one of the most important engineering and environmental issues of arid countries. The location of Kuwait is appropriate for being a case study as the country has high solar potential, high cooling load and high dependence on desalinated water. In addition to the well-established thermal desalination, seawater reverse osmosis (SWRO) is currently one of the most significant desalination technologies due to its high energy efficiency compared to the thermal desalination process; however, the energy cost of the pressure-driven separation process is a major limitation [1,2,3]. Recent reviews also reveal that the current state of the art of RO development is now heading towards high-permeability membranes, improved fouling management, energy recovery, artificial intelligence (AI)-based operation, and integration with renewables [4,5].

PV-RO can decrease the need for fossil-fuel electricity. PV energy is, however, intermittent, and the RO process is sensitive to feeding pressure, flow rate, temperature, salinity and membrane condition. The operation is more complex than a fixed-pressure operation, due to these variables. Renewable-powered RO, therefore, should be investigated as a water-energy system, not simply as a membrane separation unit [6,7,8,9,10]. During variable-pressure operation, the plant could be subjected to ramping, sinusoidal or irregular feeding pressure. Dynamic behavior is significant as the renewable generation and hydraulic buffers could affect the feeding pressure and flow rates during operation [11].

In this study, the term digital-twin-assisted refers to an offline virtual representation of the PV-RO process that integrates process data, predictive models, physically motivated indicators, and an operating-point ranking layer. The proposed framework does not include real-time bidirectional communication, continuous model updating, or closed-loop actuation. Therefore, it should be regarded as a digital-twin-assisted decision-support prototype rather than a fully deployed operational digital twin. Nevertheless, the framework establishes the predictive and decision-support components required for future integration with live plant data and supervisory control [12,13]. Compared with a conventional predictive model, the digital-twin-assisted framework combines process representation, prediction, and operating-point screening within a unified decision-support workflow.

This is where machine learning (ML) can help: RO behavior is nonlinear. Permeate flow, salt rejection and SEC are influenced by the interaction of various factors, including feed pressure, feed flow rate, feed conductivity, feed temperature and membrane condition. Artificial neural networks, Gradient Boosting, support vector regression, Random Forests, and explainable AI are the modeling techniques employed in recent studies to describe membrane flux, fouling, water quality, and desalination energy [14,15,16,17,18].

Most previous studies focus on steady-state operation or isolated prediction tasks. This study addresses this gap through a transparent framework that combines Kuwait solar-availability information with dynamic RO experiments, predicts freshwater production, SEC, and permeate conductivity, and ranks candidate operating points.

In terms of membrane science, the key issue is how transitory hydraulic forces affect the membrane-process results such as permeate flux, recovery rate, salt rejection, SEC, and permeate conductivity. Hence, the proposed digital twin can be considered a decision-support tool for membrane processes, not just any machine learning or renewable energy forecasting tool. The scope limitations associated with the hybrid data coupling and simplified PV representation are discussed in Section 5.6.

### 1.1. Research Objectives

1. To build a hybrid dataset combining Kuwait solar-availability information with dynamic RO experimental run.
2. To calculate process-informed features including estimated pump power, recovery, salt rejection, SEC, pressure rate, and PV power margin.
3. To train and compare Gradient Boosting, Random Forest, and XGBoost models for permeate flow rate, energy consumption, and permeate conductivity prediction.
4. To develop a multi-criteria ranking score for identifying candidate operating conditions with higher permeate flow and lower estimated energy demand.
5. To compare the proposed method with existing PV-RO, dynamic RO, machine learning, and digital-twin studies.

### 1.2. Main Contributions and Novelty

- A hybrid decision-support framework combines Kuwait solar-availability information with dynamic RO experimental behavior.
- Instead of just steady-state RO points, dynamic experimental profiles are used.
- Not a blackbox-only approach as process-informed variables are introduced prior to model training.
- The modeling of freshwater, energy, and water quality as separate outputs is discussed honestly, and there is recognition of the different levels of predictability of these outputs.
- A transparent multi-criteria ranking layer is used to identify favorable candidate operating conditions.

## 2. Literature Review

### 2.1. Reverse Osmosis Desalination and Energy Demand

RO desalination technology can be used to remove water from the dissolved salts by applying pressure higher than the osmotic pressure. It has widespread applications due to its ability to not undergo a phase change and therefore requires less energy than traditional thermal systems. According to current reviews, RO has emerged as the preferred method in numerous desalination applications, but energy usage, membrane fouling, management of the brine stream and feed-water variability are still significant constraints [1,5,19,20,21].

Specific energy consumption is one of the key performance measures for RO operation. Salinity, pressure, recovery ratio, flow rate, pump efficiency and energy recovery all have an impact on it. Recent research on SWRO minimum energy and SEC modeling reveals potential for further efficiency improvements, which will necessitate improved system-level control, process integration and monitoring using data [2,22,23]. In this context, SEC prediction is not just a reporting metric, it is also an optimization target for digital-twin control.

### 2.2. Renewable and PV-Powered RO Desalination

Desalination using renewable energy is appealing in areas with high demand for freshwater and good solar-energy availability. Solar PV-RO is one of the most viable combinations suggested by reviews on renewable desalination, as PV electricity can directly drive pumps and auxiliary equipment [6,7,24]. Small and remote systems have also been explored for the use of solar PV-RO, such as in regions with high extension costs or where diesel generation is not desired [25,26].

However, there are two interrelated issues with PV-RO systems. PV output varies with irradiance, temperature, time of day, season and weather. Kuwait-based photovoltaic studies have also shown that air-pollution effects, solar irradiation, and tilt-angle selection can strongly influence PV-system performance and economic operation [27,28,29,30,31,32]. Secondly, RO membranes are sensitive to the changes in hydraulic conditions, and, thus, uncontrolled changes in hydraulic conditions can affect salt rejection or performance. Batteries, pressure accumulators, energy recovery and control strategies for stabilizing operation have thus been studied [8,27,28,29,33,34]. This challenge is used as motivation in the present study for a data-driven digital twin that can estimate the impact of operating pressure and flow conditions prior to actual operation. Beyond PV-powered RO, low-grade thermal-energy recovery offers another pathway for sustainable desalination, including thermoelectric-assisted membrane systems that combine energy recovery with freshwater production [35].

### 2.3. Dynamic RO Operation Under Variable Feed Pressure

Steady operating conditions are assumed for most classical RO models, but renewable energy can cause transient feed pressure and flow. This is particularly true for those systems that are powered by waves, such as wave-powered systems or direct-drive systems, where the pressure waves, accumulators, and hydraulic conversion may result in a ramping or sinusoidal input [34,36,37]. Dynamic RO experiments are therefore useful since they demonstrate the behavior of membranes under non-static pressure and flow conditions. The NREL experimental wave desalination dataset is very relevant to this paper. It gives raw data for steady-state, trapezoidal ramping, sinusoidal and WEC-Sim waveform experiments.

According to Data.gov, the dataset was designed to characterize RO membrane performance under dynamic conditions of feed pressure and flow rate, and that the files are raw CSV data without any additional processing to remove startup and shutdown data [38]. The dataset also includes a process-flow diagram of the experimental system, which is used and referenced in this manuscript.

### 2.4. Machine Learning for Membrane and Desalination Systems

The use of machine learning for membrane desalination is growing due to its ability to model complex, nonlinear relationships without the need to solve all transport equations explicitly. In recent years, AI has been utilized in various areas, including flux prediction, fouling detection, water quality estimation, operating condition optimization, and predictive maintenance [4,14,15,39].

The studies all seem to agree that data quality, correct feature selection and interpretability are vital for successful deployment. Artificial neural networks (ANN), Random Forests, support vector methods, Gradient Boosting and explainable AI techniques are some of the more recent methods used in the literature for RO-specific modeling. Karimanzira and Rauschenbach [40] demonstrated the potential of using ML for RO performance prediction. In this regard, Mohammed et al. [17] highlighted the need for holistic frameworks for RO prediction, and Alizamir et al. [16] employed explainable approaches for flux prediction. Gao et al. [18] applied ML to optimize the membrane material and performance. The studies pointed in favor of the selection of tree-based ensemble models, and this is done in the present work particularly for tabular experimental data [41,42,43]. Popular methods are XGBoost and Gradient Boosting methods, since they are able to capture nonlinear interactions and deal with mixed features well [44]. Random Forest-type models are also appropriate for tabular process data since they are resistant to nonlinear relationships and noisy variables. The machine-learning stage was designed as a transparent and reproducible pipeline rather than a complex black-box model.

### 2.5. Digital Twins in Water Treatment and Desalination

Digital-twin concepts have extended from manufacturing to water treatment, wastewater treatment, and desalination. The data acquisition, virtual modeling, prediction, feedback and decision support are typically included in a digital twin. The water sector’s latest water sector reviews demonstrate the potential of digital twins for forecasting, optimization, predictive maintenance, and operational decision-making [12,13].

Digital twins are particularly valuable in desalination, where energy costs and reliability of the process are key. The use of digital twins and ML for enhancing the efficiency of SWRO has been investigated by Daaboub et al. [45], and recent water sector reviews have found that decisions around RO energy, fouling, cost, and cleaning are potential digital-twin applications [13,46], but there are several published digital twin discussions that are either conceptual or plant specific. The novelty in this is the integration of open datasets, Kuwait Solar data, dynamic RO experiments, process-informed feature engineering, and selection layer in a single transparent workflow.

### 2.6. Research Gap

The literature shows five main gaps. First, many PV-RO studies focus on system sizing or steady-state performance, while fewer use dynamic pressure experiments. Second, many ML studies predict one process output, but they do not connect prediction to PV energy availability and optimization. Third, digital-twin studies often require private plant data, which limits reproducibility. Fourth, water quality prediction is often treated as equal to flow prediction, although quality may require richer chemical and membrane-state inputs. Fifth, few studies explicitly cite and integrate open process-flow images, raw dynamic RO data, and local solar data in a complete manuscript-ready workflow. The proposed study was designed to address these gaps in a realistic but reproducible way.

## 3. Comparison with Existing Studies

To contextualize the contributions of this work within the broader research landscape, the proposed digital-twin framework is systematically compared against selected landmark studies from the literature. A granular breakdown of this landscape across system focus, methodological taxonomy, core contributions, and key differentiators is summarized in Table 1.

## 4. Materials and Methods

### 4.1. Study Area, Data Sources, and Source Citation

Kuwait City was selected as the applied case-study location. The solar and meteorological variables were obtained from NASA POWER using the hourly application programming interface (API). NASA POWER describes the hourly API as a time-series service for solar and meteorological data that can be used directly by applications [47]. NASA POWER hourly data for Kuwait City (29.3759° N, 47.9774° E) covering January 2024–December 2025 were obtained. Variables included global horizontal irradiance (GHI), ambient temperature, wind speed, and relative humidity. The NASA POWER and NREL datasets were not physically synchronized or treated as co-measured observations from the same plant. Instead, NASA POWER data were used to represent hourly solar-energy availability in Kuwait, whereas the NREL dataset was used to characterize the dynamic hydraulic and membrane response of the RO process. The two datasets were therefore coupled at the scenario level to provide a hybrid virtual assessment framework rather than field validation of an operating Kuwait PV-RO plant. The Photovoltaic Geographical Information System (PVGIS) was also reviewed as a supporting source because its hourly radiation tool provides solar radiation and PV power calculation data for long time periods and supports comma-separated values (CSV) and JavaScript Object Notation (JSON) download [48,49]. The implications of this hybrid data-coupling approach are further discussed in Section 5.6.

**Figure 1:**
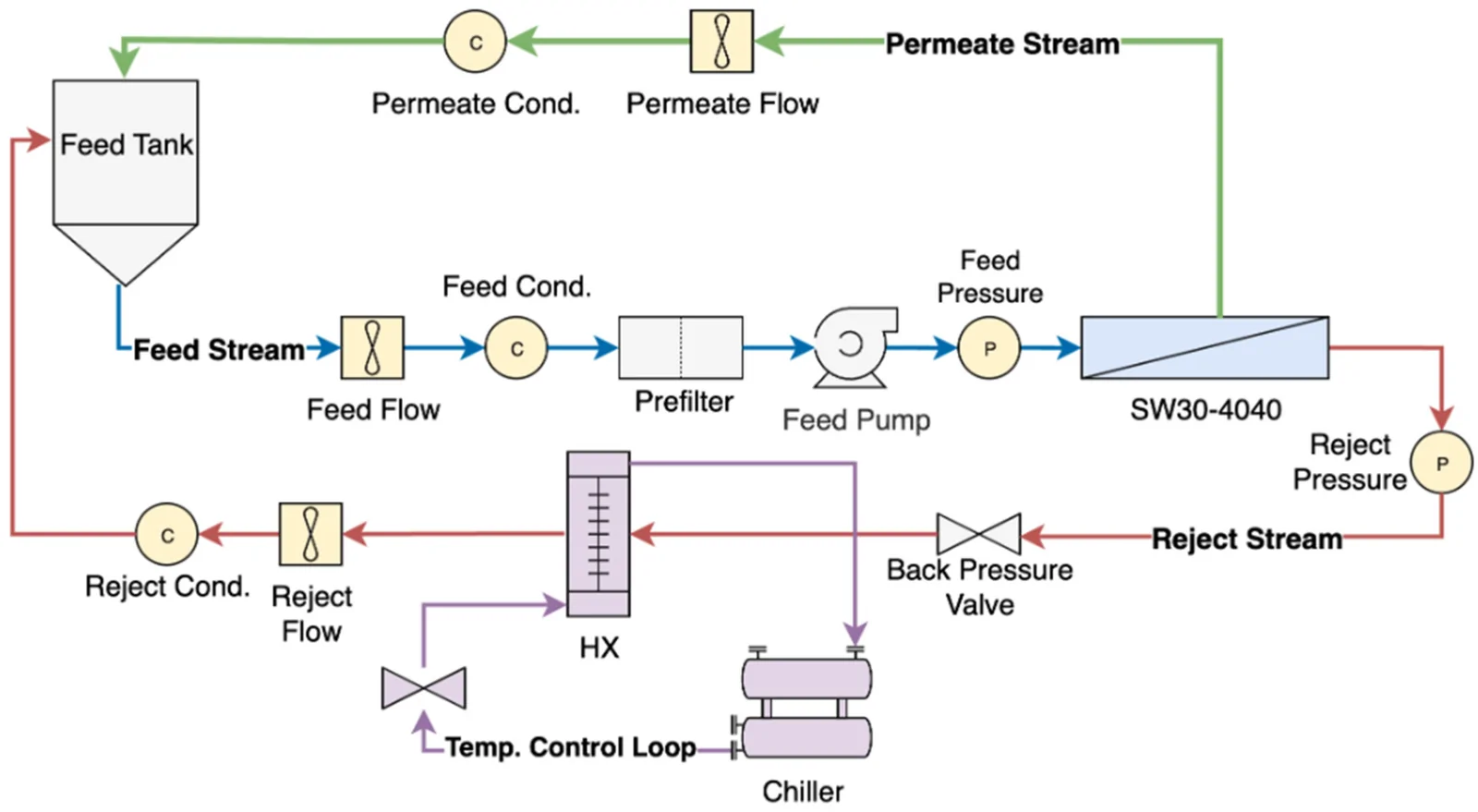
Experimental RO process-flow diagram used to explain the source RO system. Reproduced from the National Renewable Energy Laboratory/Marine and Hydrokinetic Data Repository dataset “Experimental Wave Desalination Data with Variable Feed and Pressure Input” under the Creative Commons Attribution 4.0 International (CC BY 4.0) license [38].

The RO experimental data were obtained from the NREL/U.S. Department of Energy (DOE) experimental wave desalination dataset. The dataset page states that the experimental runs were collected to characterize RO membrane performance under dynamic feed pressure and flow conditions, and that four experiment types are included: steady-state, trapezoidal ramping, sinusoidal, and WEC-Sim waveform data. The configuration of the experimental RO system used to generate the dynamic pressure and flow datasets is shown in Figure 1. The process-flow diagram illustrates the feed, membrane, permeate, and reject streams employed in the original NREL experimental setup.

### 4.2. Dataset Structure and Preprocessing

**Figure 2:**
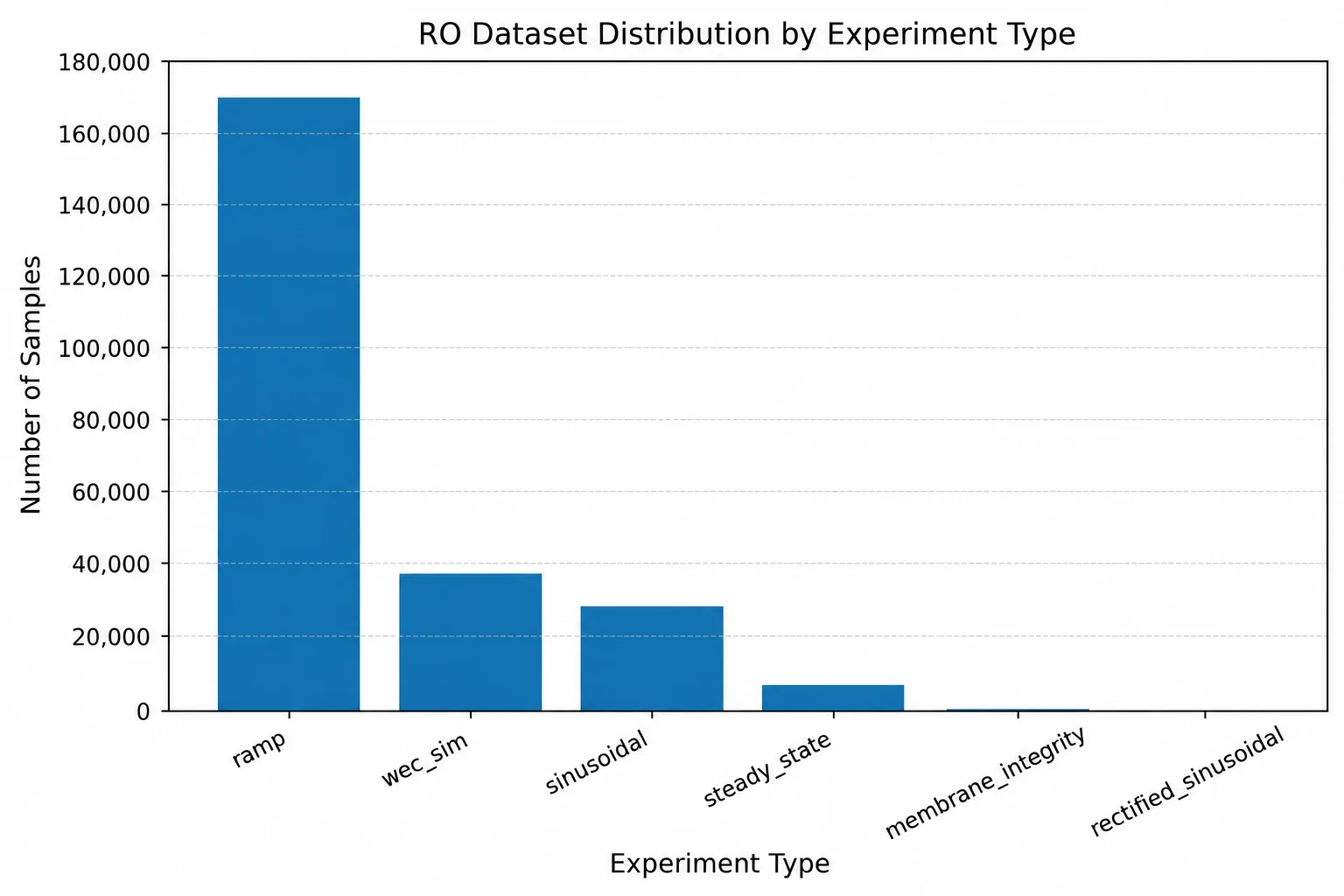
Distribution of records across RO experiment types based on the NREL dataset.

The modeling dataset combines dynamic RO experimental run with PV and meteorological information at the scenario level. In particular, the RO dataset includes several experimental runs based on the NREL wave desalination repository. This dataset includes experiments performed under steady-state conditions, ramp conditions, sinusoidal conditions, rectified sinusoidal conditions, membrane integrity conditions, and WEC-Sim operations. The distribution of the dataset based on experiment types is provided in Figure 2, wherein ramp and WEC-Sim experiments constitute the bulk of experiments available in the database. More than 240,000 entries represent sequential time-step experimental runs or observations from experimental operating categories; they should not be interpreted as 240,000 independent experiments.

As part of preprocessing, all raw CSV and spreadsheet files were converted into a single unified format. Moreover, variable names were standardized so that consistent terminologies could be used across the dataset. Standardized variables include feed pressure, feed flow rate, permeate flow rate, permeate conductivity, feed conductivity, feed temperature, reject flow rate, water flux, and experiment type. Only complete duplicate rows were removed; repeated sensor values were retained because they may represent legitimate sequential measurements.

A preliminary evaluation of the dataset indicated significant differences between the number of observations in each experiment type. As shown in Table 2, ramp tests constituted the highest portion of the dataset followed by WEC-Sim tests and sinusoidal tests. By comparison, membrane integrity and rectified sinusoidal experiments formed only a small fraction of the dataset. Nonetheless, all experiments were maintained since they represent different hydraulic operation modes.

A complete case approach was applied for each prediction target: experimental runs lacking the variables required for the corresponding model were excluded, and no missing target values were imputed or synthetically generated. Experimental runs with SEC above 200 kWh/m^3^ were excluded as a numerical-stability screening step because SEC becomes extremely sensitive when permeate flow approaches zero. This threshold is not presented as a physical plant-operating limit or proof that all excluded experimental runs were erroneous. Overall, more than 240,000 experimental runs remained after cleansing and formatting; from these, the representative subset 60,000-experimental run described in Section 4.4 was used for model development. Statistical parameters for process variables of the dataset are given in Table 3. Mean feed pressure amounted to 745.39 psi, whereas mean feed flow rate and permeate flow rate were 21.33 L/min and 2.76 L/min, respectively. Furthermore, the mean specific energy consumption was 17.85 kWh/m^3^. Mean recovery ratio and salt rejection values reached 12.41% and 99.43%, respectively.

Since the data for NREL was taken in controlled conditions of the membrane test, the interpretation of the dataset was mainly that of a RO membrane transient response dataset. Thus, full-scale design values could not be derived, only potential operating regions.

### 4.3. Feature Engineering

The digital-twin dataset included both measured and derived variables. The measured variables included feed pressure, feed flow rate, feed conductivity, feed temperature, permeate flow rate, permeate conductivity, reject flow rate, water flux, and experiment type. Derived variables included pressure difference, absolute pressure difference, recovery ratio, salt rejection, pump power, SEC, pressure rate, rolling means, rolling standard deviations, and one-step lag values. These features were used because they represent physical relationships in RO transport and because previous ML studies recommend using process-informed variables to reduce purely black-box behavior [15,16,17].

Feature engineering was performed to capture both the steady-state and dynamic behavior of the RO system. While the measured variables describe the instantaneous operating conditions, the derived variables provide additional information regarding process efficiency, temporal variation, and membrane performance. In particular, rolling statistics and lag features were included to capture short-term operational dynamics that may influence freshwater production, energy consumption, and water quality under variable-pressure conditions.

The main equations used during feature engineering were:

(1) Recovery (%) = (Qp/Qf) × 100

(2) Salt rejection (%) = (1 − Cp/Cf) × 100

(3) Pump power (kW) = (ΔP × Qf)/η

(4) SEC (kWh/m^3^) = Pump power (kW)/Permeate flow rate (m^3^/h)

(5) PV power margin (kW) = Estimated PV power (kW) − Estimated pump power (kW)

where Qp is permeate flow rate, Qf is feed flow rate, Cp is permeate conductivity or salinity, Cf is feed conductivity or salinity, ΔP is operating pressure, and η is the pump efficiency. A constant value of η = 0.68 was used as a fixed engineering assumption because pump-specific efficiency data were unavailable in the source dataset. In Equation (3), ΔP represents the available operating-pressure proxy. In this study, feed pressure and operating pressure refer to the same available source-dataset variable, while pressure difference and absolute pressure difference are derived descriptors and are not interpreted as sensor-resolved transmembrane pressure because detailed sensor-location metadata were unavailable. The calculated value is an estimated hydraulic pump-power indicator and excludes detailed hydraulic losses, motor efficiency, inverter efficiency, storage losses, and auxiliary electrical loads. Salt rejection is interpreted as an apparent conductivity-based indicator. No detailed ionic-speciation correction or independent conductivity-to-salinity conversion was available, and independent temperature compensation could not be verified from the processed dataset.

The PV power margin was used as an indicator of solar-energy availability and was not applied as a plant-level feasibility constraint in the optimization. The present framework does not directly map solar variability to feed pressure changes; PV data are used as a scenario-level energy-availability indicator. Accordingly, “freshwater production” in this study refers to instantaneous laboratory permeate flow rate in L/min, not daily or annual PV-driven water production, nighttime operation, seasonal yield, storage requirements, or production per installed PV capacity.

Recovery ratio and salt rejection were included because they are widely used indicators of membrane separation performance, while SEC and pump power represent key energy-performance metrics for desalination systems. The pressure-rate and pressure-difference features were introduced to quantify the transient hydraulic behavior observed in ramp, sinusoidal, and WEC-Sim experiments. Similarly, lag variables and rolling statistics were generated to provide the machine-learning models with information regarding recent operating history, thereby improving their ability to capture dynamic process responses [37]. Detailed rolling-window duration and run-boundary implementation records were unavailable for independent reporting; therefore, rolling and lag variables are treated only as historical process descriptors.

The engineered variables also provide a membrane-specific interpretation layer: pressure rate and rolling pressure describe hydraulic transients; recovery and salt rejection describe separation behavior; SEC and pump power describe energy penalty; and permeate conductivity represents the water quality endpoint. This mapping helps readers connect the machine-learning inputs to membrane-process mechanisms.

These equations form the process-informed layer of the proposed decision-support framework [2,23]. Here, “process-informed” refers to engineered variables derived from established RO relationships; no physical constraints were imposed during model training.

### 4.4. Machine Learning and Model Implementation

The machine-learning component of the proposed framework was designed to predict three core process variables, namely, permeate flow rate, SEC, and permeate conductivity. Following feature engineering, a representative subset of 60,000 records was selected to preserve representation of the available experiment types and operating ranges. This subset also reduced redundancy in the densely sampled sequential measurements and enabled a consistent comparison of the three models. To improve transparency, Table 4 distinguishes measured and derived variables and identifies variables that are mathematically related to each prediction target.

To predict the three process variables in question, three supervised ensemble algorithms were considered: Gradient Boosting Regressor, Random Forest Regressor, and XGBoost (Extreme Gradient Boosting). These algorithms have been demonstrated as powerful predictors in nonlinear engineering datasets, as well as being able to handle tabular data involving intricate interactions between process features. Moreover, tree-based ensemble models provide high predictive power with low dependency on the underlying distribution of data.

In this study, a set of process-related features discussed in Section 4.3 was used as predictors for each of the above-mentioned target process parameters. Specifically, separate models were trained for predicting each target process variable in order to independently evaluate the models’ predictive capabilities. To improve model interpretability, the relevance of the principal operational variables was examined based on the observed process trends and the physical relationships represented by the engineered features.

To minimize data leakage from sequentially correlated observations, the data were partitioned at the source experimental-dataset level rather than at the individual-record level. All experimental runs originating from the same source dataset were assigned exclusively to either the training or held-out test partition, ensuring that correlated observations were not shared between the two partitions. The reported evaluation should therefore be interpreted as an internal source-dataset-level holdout assessment; no stronger claim of strict run-level or external validation is made.

Accordingly, the reported MAE, RMSE, and R^2^ values represent internal holdout validation within the available NREL source dataset and should not be interpreted as external, pilot-scale, or field validation of the proposed framework.

Prediction performance was evaluated using mean absolute error (MAE), root mean square error (RMSE), and the coefficient of determination (R^2^). MAPE was excluded from permeate flow evaluation because permeate flow values approaching zero make percentage-based errors numerically unstable and non-informative. All three models were evaluated on the same held-out test partition to ensure a consistent comparison of their predictive performance.

The final selection of models relied on their predictive accuracy and generalization performance. As such, Gradient Boosting provided superior accuracy for permeate flow prediction, whereas Random Forest was the best performer among the other two algorithms when applied to SEC and permeate conductivity prediction. Consequently, the selected algorithms were implemented in the decision-support framework for further analysis.

The modeling strategy was intentionally designed to prioritize transparency, reproducibility, and practical engineering applicability. Consequently, the study focused on established ensemble-learning methods rather than highly complex deep-learning architectures, which typically require larger datasets, greater computational resources, and more extensive hyperparameter tuning. This approach provides a transparent basis for future extension of the framework.

### 4.5. Weighted Multi-Criteria Scoring and Operating-Point Selection

The selected models were subsequently used to calculate a weighted multi-criteria score, rank the available operating points, and retain the top 20 candidate conditions. Following prediction, the best-performing models were used as the predictive core of the digital-twin-assisted decision-support framework. The present implementation represents the predictive and decision-support layers of digital-twin architecture, while real-time sensing, continuous updating, and closed-loop control remain outside the scope of this study. A weighted multi-criteria ranking score was used to evaluate the candidate operating points. Permeate flow rate was rewarded, whereas SEC and permeate conductivity were penalized. Prior to score calculation, all prediction outputs were normalized to ensure that variables with different units and magnitudes contributed proportionally to the scoring process. The weighted multi-criteria score was determined as follows:

(6) weighted multi-criteria ranking score = 0.50 × permeateflowrate_norm − 0.35 × SEC_norm − 0.15 × quality_norm

The weights were selected to reflect the primary objective of the proposed decision-support framework, namely favoring higher instantaneous permeate flow while reducing estimated energy consumption and maintaining acceptable water quality. Greater emphasis was therefore assigned to freshwater production and SEC, while permeate conductivity was treated as a supporting water quality indicator. The weighted multi-criteria ranking approach was selected because the study evaluates a finite set of candidate operating points rather than searching a continuous design space. Unlike formal Pareto-based methods, it provides a transparent screening of the available operating conditions and is therefore used as a decision-support ranking tool rather than a global optimizer [50,51]. Accordingly, the fixed weights were retained as engineering-priority coefficients, and the selected operating points are interpreted as favorable candidates rather than unique preferred candidate conditions. Because no formal weight-sensitivity analysis was performed, the ranking should be interpreted as conditional on the selected engineering-priority weights.

The weighted multi-criteria ranking score is meant to be used for screening purposes rather than as an individual control law. The potential operating points will have to comply with membrane manufacturers’ pressure limitations, pretreatment guidelines, scaling and fouling thresholds, cleaning restrictions, and permeate-quality specifications prior to execution.

Operating points were selected as the top 20 candidate solutions according to the weighted multi-criteria ranking score. These operating points represent conditions that simultaneously achieve higher permeate flow rate and lower energy consumption relative to the broader candidate space. The selection layer is not intended to function as a real-time plant controller, but rather as a repeatable decision-support mechanism that can identify favorable operating regions for further analysis.

The resulting weighted scoring and selection framework demonstrates how predictive machine-learning models can be integrated into an offline digital-twin-assisted decision-support framework to support operational decision-making. Furthermore, the proposed approach is computationally simple and expandable to more advanced optimization strategies, including constrained optimization and model predictive control, in future work. Related constrained predictive-control studies further support the use of prediction-based control layers for dynamic engineering systems [28,51,52].

## 5. Results and Discussion

### 5.1. Dataset Behavior and Physical Interpretation

There were multiple types of experiments in the dataset, with the largest amount of data being from ramp experiments, followed by WEC-Sim and sinusoidal experiments (Table 2 and Figure 2). The average feeding pressure was 745.39 psi and the standard deviation was 147.57 psi. The average permeate flow rate was 2.76 L/min, and the average SEC after the elimination of outliers was 17.85 kWh/m^3^ (Table 3). These values indicate that the dataset is not a full-scale optimized SWRO plant dataset, but rather a laboratory dynamic membrane dataset. Thus, the results should be seen for digital-twin method validation and operational trend analysis, and not as design performance at the plant scale.

**Figure 3:**
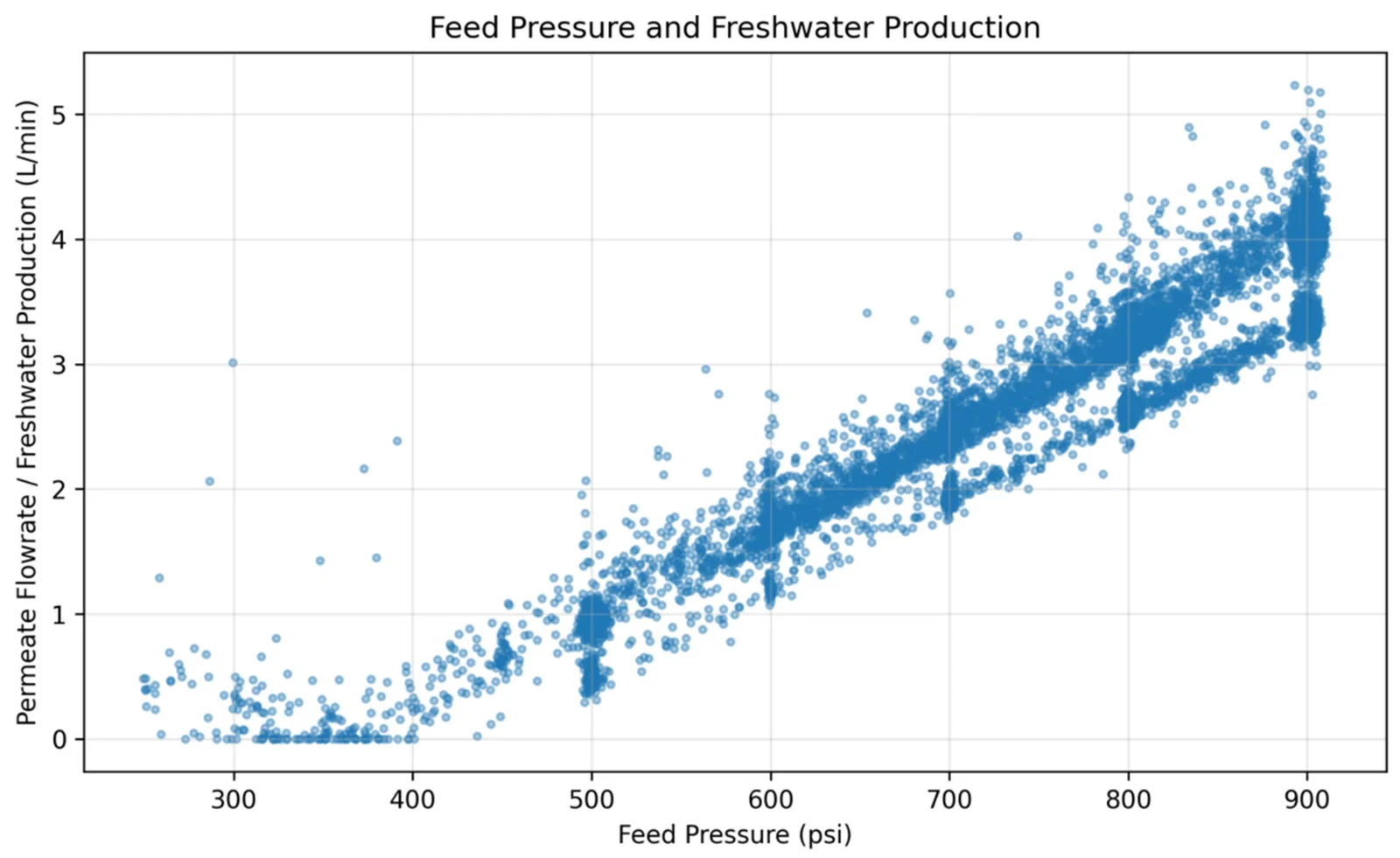
Feed pressure and permeate flow rate relationship in the prepared RO dataset.

It can be seen from Figure 3 that there is a good positive relationship between feed pressure and permeate flow rate. This is consistent with RO transport theory as applied pressure will increase the driving force for water flux, but the patterns of scatter and bands indicate that pressure alone is not enough. The final output of freshwater is affected by the feed flow rate, the type of waveform, water flux and the type of source experiment. This result reinforces the need for a multivariable machine-learning model (not just a single pressure–flow correlation).

The pressure–flow relationship explains why the optimum is not merely the highest pressure. Higher pressures improve flux, but they can lead to increased compaction force, higher energy requirements, and increased vulnerability to scaling/fouling; hence, the digital twin has to take into account energy costs as well as gains in flux.

**Figure 4:**
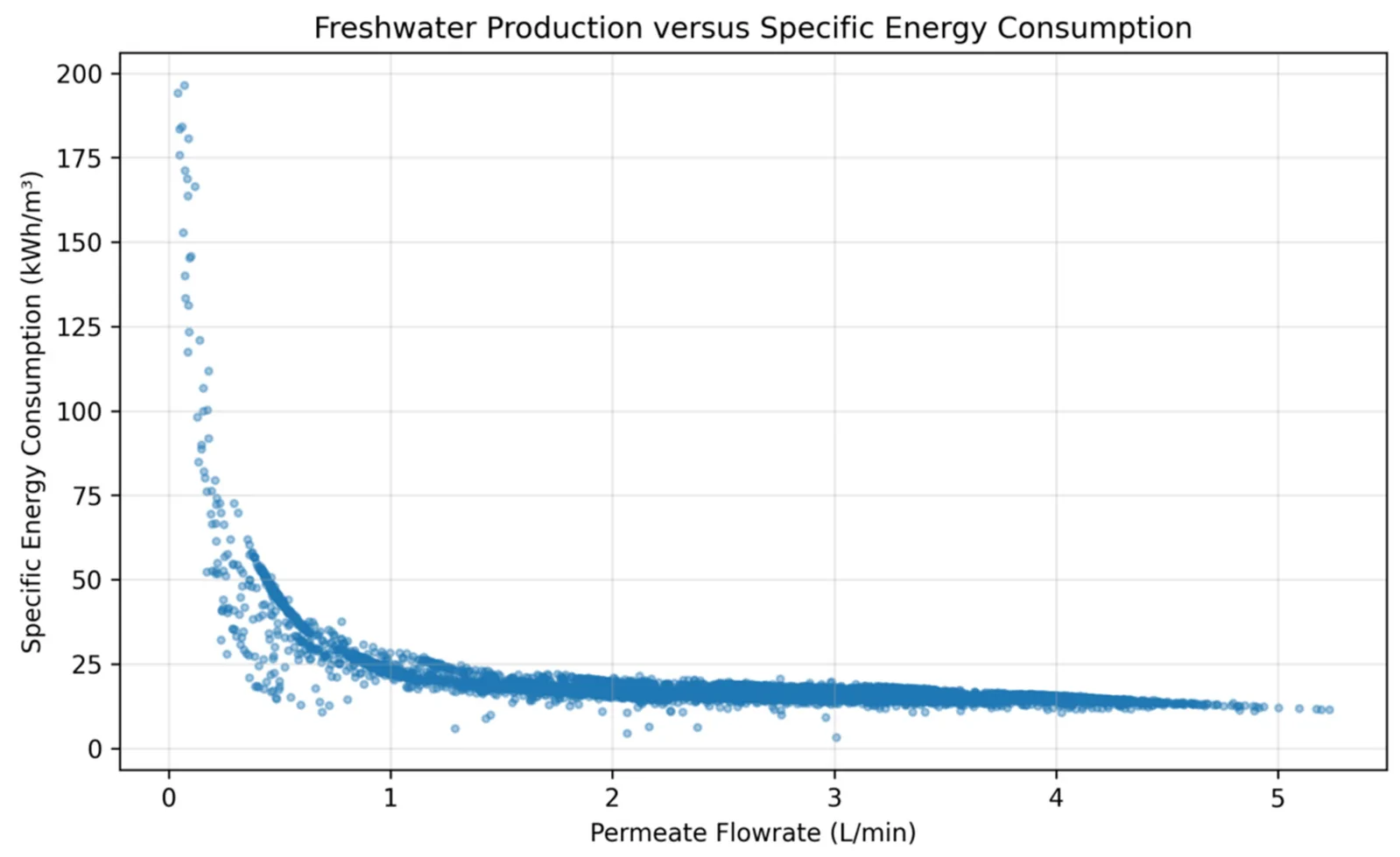
Permeate flow rate versus specific energy consumption.

The general trend for SEC is that higher production rates correspond to lower SEC values, particularly at low production rates, as seen in Figure 4. The high SEC tail is primarily due to the low permeate flow rate since SEC is defined as the ratio of pump power to the amount of permeate produced. This accounts for the fact that energy prediction proved to be harder than freshwater prediction. In low flow, both physical operation and mathematical sensitivity influence the energy target. Figure 4 illustrates the relationship between freshwater production and specific energy consumption across the investigated operating conditions.

### 5.2. Permeate-Flow-Rate Prediction

**Figure 5:**
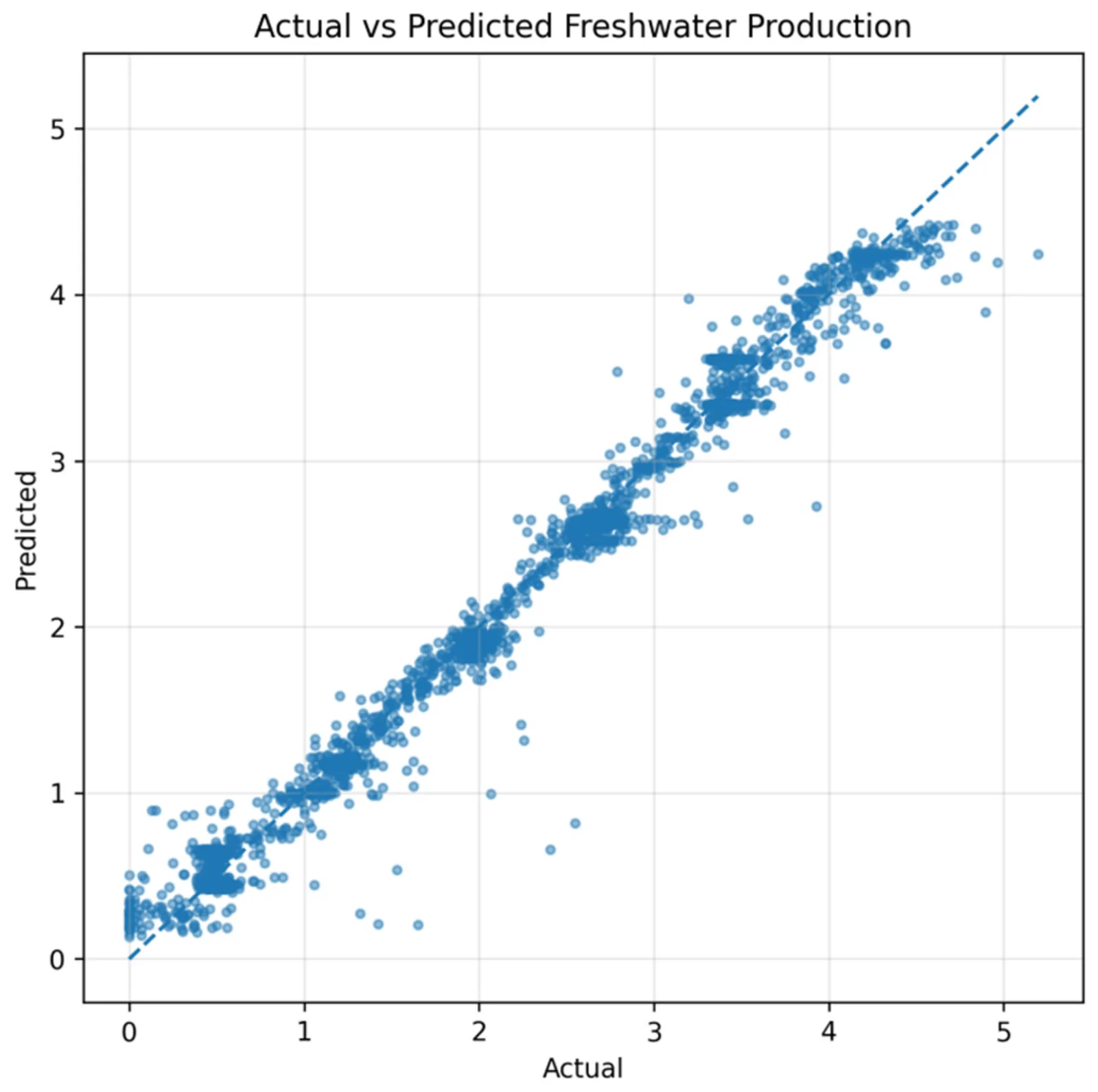
Permeate-flow-rate prediction model comparison.

**Figure 6:**
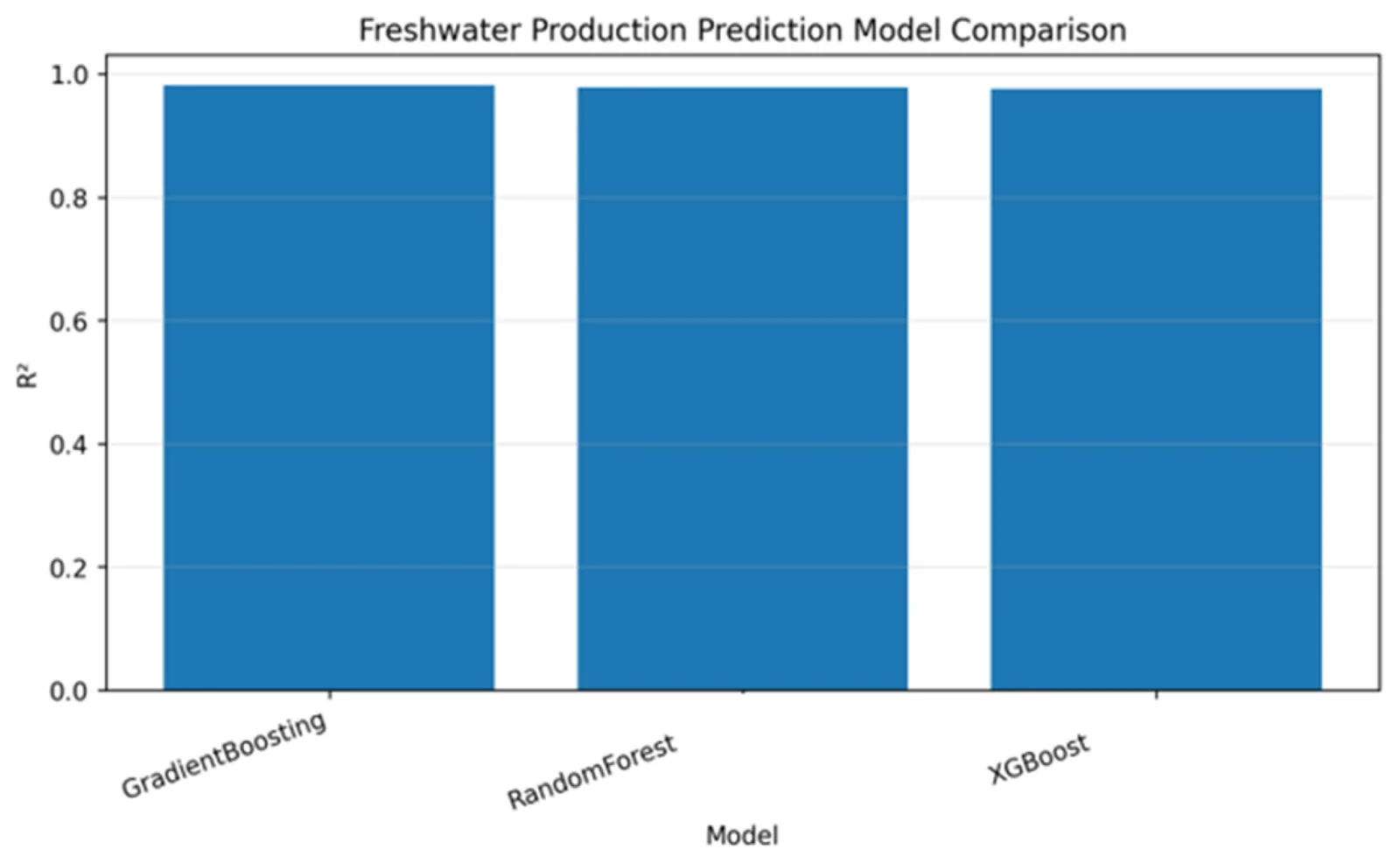
Actual-versus-predicted permeate flow rate for the best model.

The best prediction target was permeate flow rate. Gradient Boosting achieved the best performance, with MAE = 0.106 L/min, RMSE = 0.161 L/min, and R^2^ = 0.981 (Table 5). Random Forest and XGBoost also showed good results, with a R^2^ of 0.978 and 0.976 respectively. Figure 5 shows that most of the predicted values lie close to the diagonal line, indicating that the model was able to capture the general freshwater production behavior. Each scatter point represents an individual actual–predicted observation, while the dashed diagonal line represents perfect agreement between the actual and predicted values. Within the prepared NREL dataset, Gradient Boosting reproduced permeate flow patterns with an RMSE of 0.161 L/min and an R^2^ of 0.981. Because some engineered variables are mathematically related to permeate flow, this result is interpreted as internal surrogate-estimation performance rather than leakage-free independent prediction. It also corroborates the existing literature that indicates that ML is well suited to predict the flux and output of membranes when appropriate operational variables are available [4,17,39]. Figure 6 compares the performance metrics of the evaluated freshwater production prediction models.

### 5.3. Specific Energy Consumption Prediction

**Figure 7:**
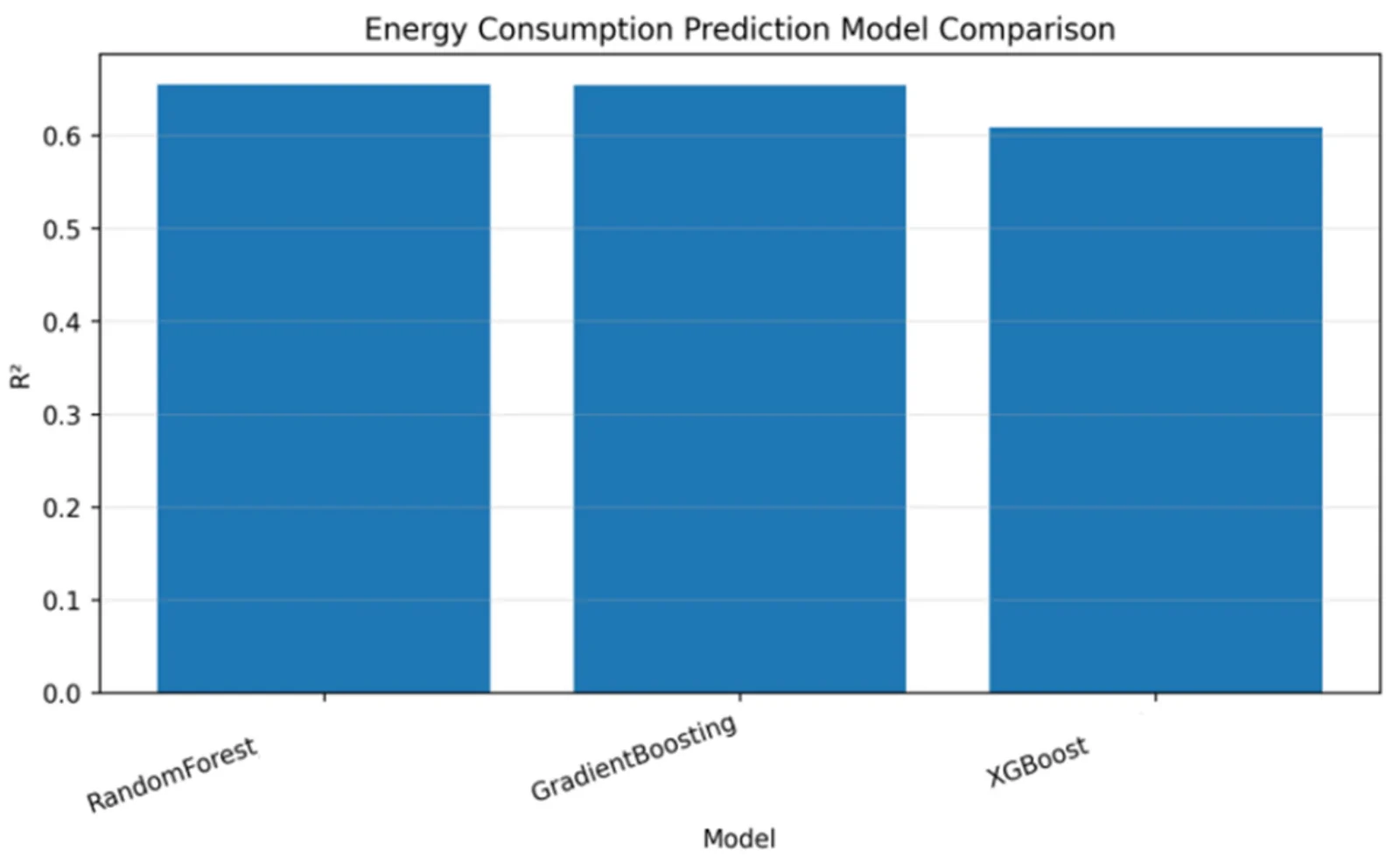
Specific energy consumption prediction model comparison.

**Figure 8:**
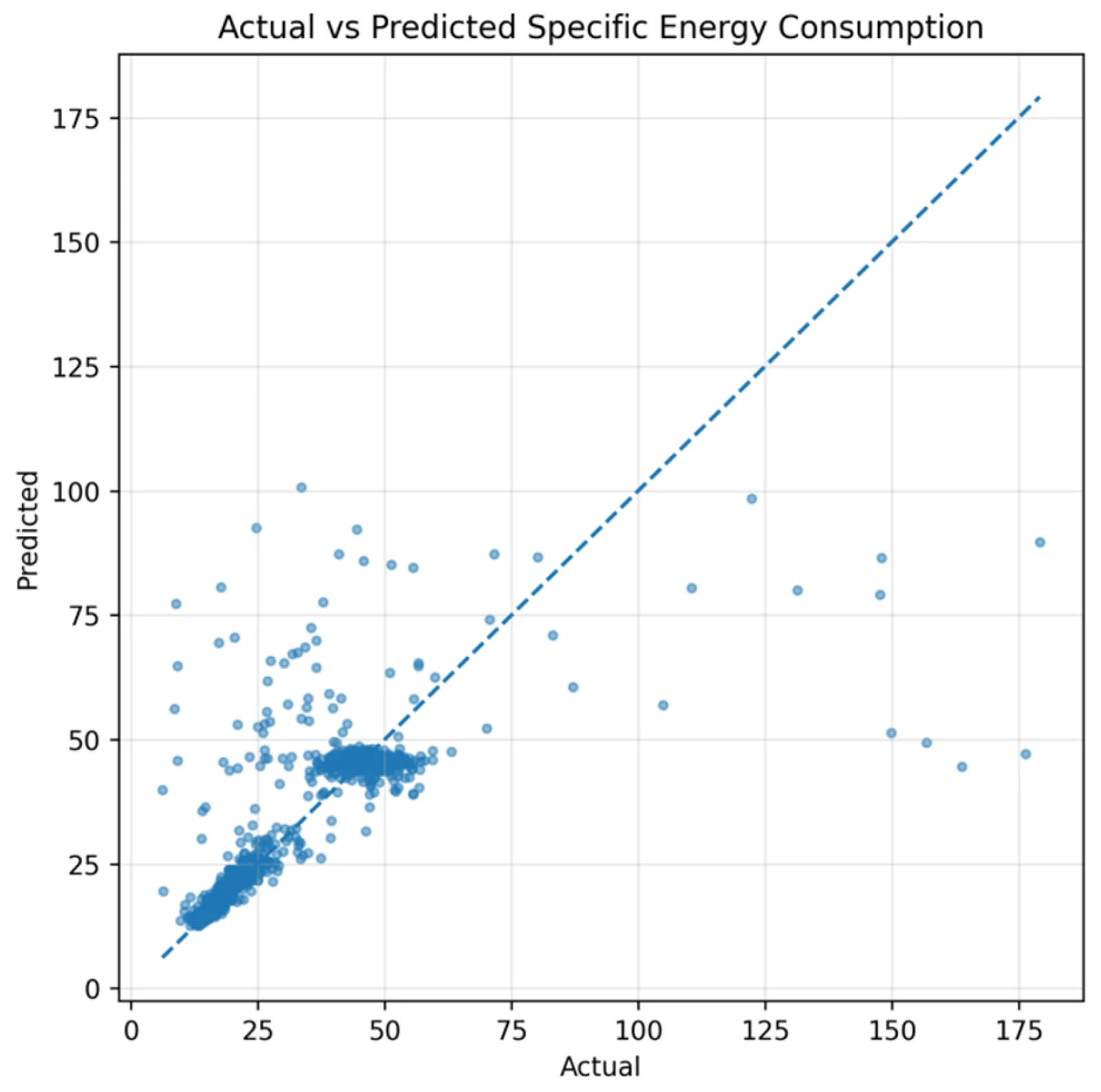
Actual-versus-predicted SEC for the best model.

Energy prediction was more difficult than freshwater prediction. Random Forest achieved the best SEC result, with MAE = 2.098 kWh/m^3^, RMSE = 7.570 kWh/m^3^, and R^2^ = 0.654 (Table 6). Gradient Boosting achieved a similar R^2^ of 0.654, while XGBoost produced R^2^ = 0.609. Figure 7 compares the predictive performance of the evaluated SEC models, while Figure 8 presents the actual-versus-predicted SEC values for the best-performing model. Each scatter point represents an individual actual–predicted SEC observation, while the dashed diagonal line indicates perfect agreement between the actual and predicted values; the wider dispersion around this line reflects the lower predictive accuracy of the SEC model. This is expected because SEC becomes unstable when permeate flow is small. It also depends on estimated pump power, which was calculated from pressure and feed flow rather than measured directly. The SEC model represents a surrogate of the calculated hydraulic SEC indicator defined in Equation (4), rather than an independent prediction of measured plant electrical energy demand. Relative to the mean SEC of 17.85 kWh/m^3^, the RMSE of 7.570 kWh/m^3^ is approximately 42%, confirming moderate quantitative accuracy, particularly under high-SEC and low-flow conditions. The model is therefore used only for operating-region screening rather than absolute SEC certification.

Although the SEC model is weaker than the permeate flow model, it remains useful for operating-region screening because it distinguishes lower- and higher-SEC regions within the analyzed dataset. This agrees with recent energy-focused SWRO studies showing that SEC analysis requires system-level variables and careful treatment of operational boundaries [2,22].

### 5.4. Water Quality Prediction

**Figure 9:**
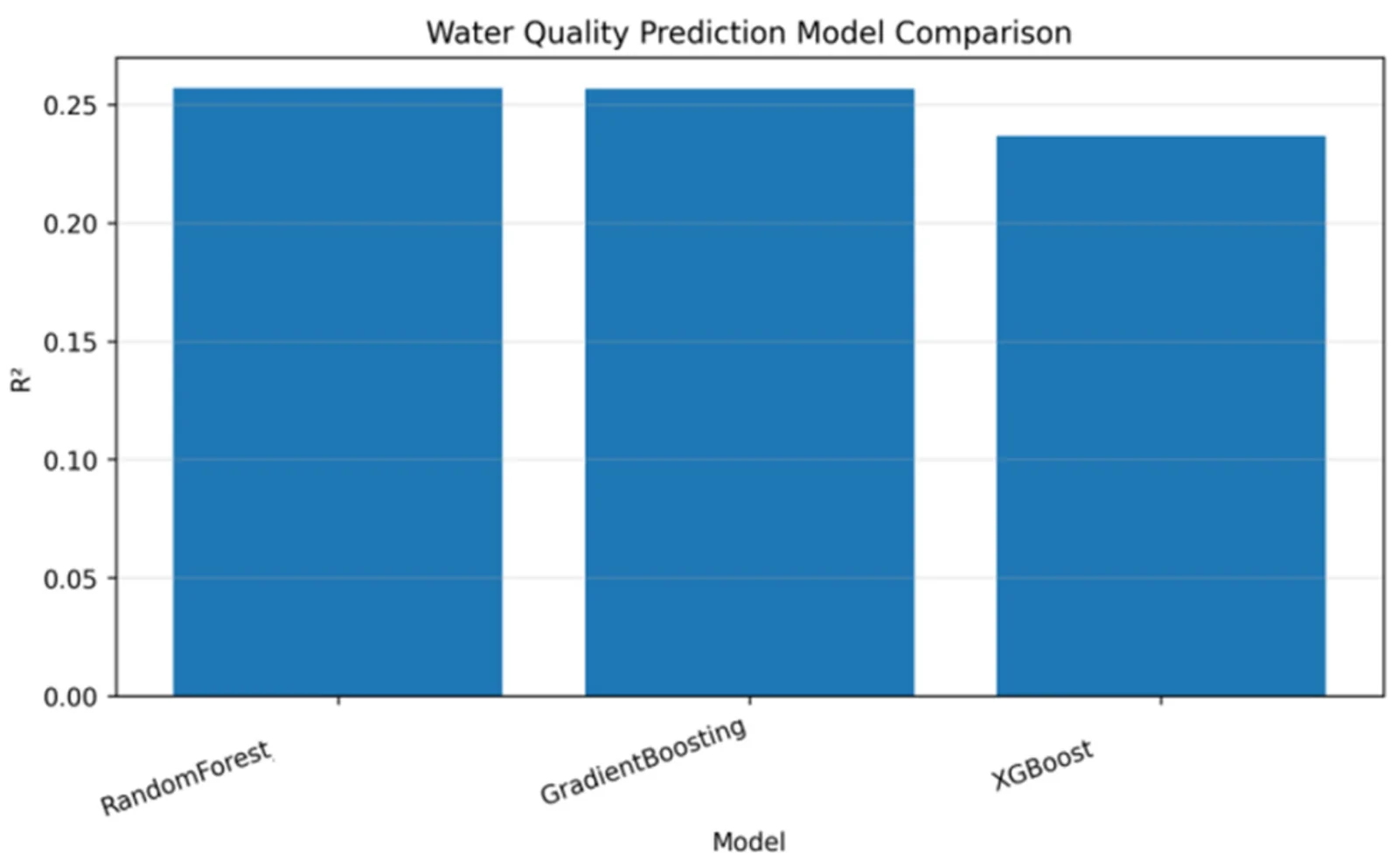
Water quality prediction model comparison.

**Figure 10:**
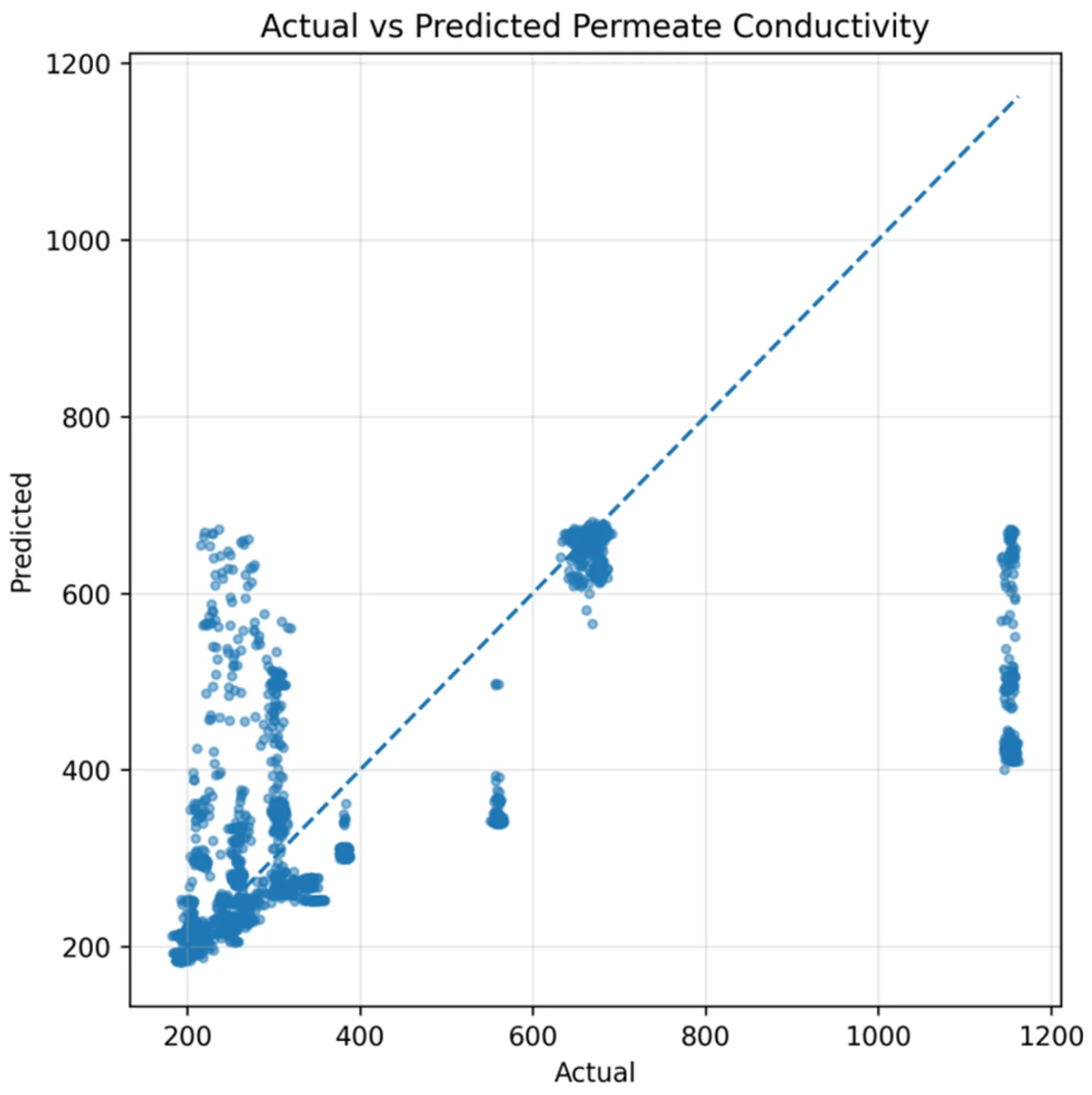
Actual-versus-predicted permeate conductivity for the best model.

Permeate conductivity was the weakest prediction target. Random Forest gave the best result, but the R^2^ value was only 0.257, and RMSE was 245.44 µS/cm (Table 7). Figure 9 compares the predictive performance of the evaluated machine-learning models for permeate conductivity prediction, while Figure 10 shows clustered predictions and clear underprediction or overprediction in some conductivity regions. Each scatter point represents an individual actual–predicted conductivity observation, while the dashed diagonal line represents perfect agreement between the actual and predicted values. This suggests that water quality prediction requires additional inputs that were not fully available in the current merged dataset, such as detailed membrane age, scaling/fouling state, osmotic pressure, ionic composition, pretreatment condition, and high-frequency sensor calibration information. The difference in mean conductivity between the selected and baseline records is smaller than the conductivity-model RMSE. Consequently, no quantitative water quality improvement is claimed, and conductivity is retained only as a low-weight secondary screening indicator.

This result is important because it prevents overclaiming. The digital twin is strong for freshwater production and useful for energy optimization, but it should not be presented as a complete water quality controller. Future work should include richer chemistry and membrane-condition data, as recommended in AI-membrane reviews and fouling prediction studies [53,54].

With respect to membrane-related research paper, the low conductivity forecast is expected to be a good diagnostic conclusion since this shows that flow and energy predictions depend on hydraulics, while for water quality predictions, more membrane conditions and solution chemistry parameters like membrane age, cleaning conditions, fouling properties, ion content, and osmotic pressure need to be considered.

The observed trends indicate that feed pressure, feed flow rate, and transient pressure descriptors are the principal operational variables associated with freshwater production behavior. Estimated pump power, feed pressure, and feed flow rate are directly related to SEC behavior. For permeate conductivity, feed conductivity and feed temperature are the most relevant available inputs; however, the limited prediction accuracy indicates that additional membrane-condition and feed-chemistry variables are required.

### 5.5. Results of Weighted Operating-Point Selection

The trained machine-learning models were integrated into the digital-twin selection layer to evaluate candidate operating conditions and identify favorable operating regions. The weighted scoring and selection framework ranked candidate points according to the weighted ranking score described in Section 4.5, which simultaneously rewards freshwater production while penalizing specific energy consumption (SEC) and permeate conductivity. Accordingly, the operating-point selection results represent the permeate flow–energy trade-off under a solar-availability context rather than a complete PV-system feasibility assessment.

Given its limited prediction accuracy, permeate conductivity was treated as a secondary screening indicator and interpreted cautiously. Its lower weight in the score limits its influence; therefore, the selected operating points mainly represent the permeate flow–SEC trade-off rather than definitive water quality optima.

Table 8 compares the average predicted performance of all candidate operating conditions (baseline) with the subset of operating points selected by the weighted scoring and selection framework. The baseline represents the full candidate operating space considered during optimization, whereas the selected conditions correspond to the highest-ranked operating points according to the weighted multi-criteria ranking score.

Compared with the full candidate set, the top-ranked subset had a mean predicted permeate flow of 4.344 L/min versus 2.750 L/min and a mean estimated hydraulic SEC of 13.69 kWh/m^3^ versus 18.50 kWh/m^3^. These correspond to descriptive differences of 57.92% in permeate flow and 25.99% in SEC, arising from the ranking procedure, and should not be interpreted as experimentally demonstrated process improvements.

**Figure 11:**
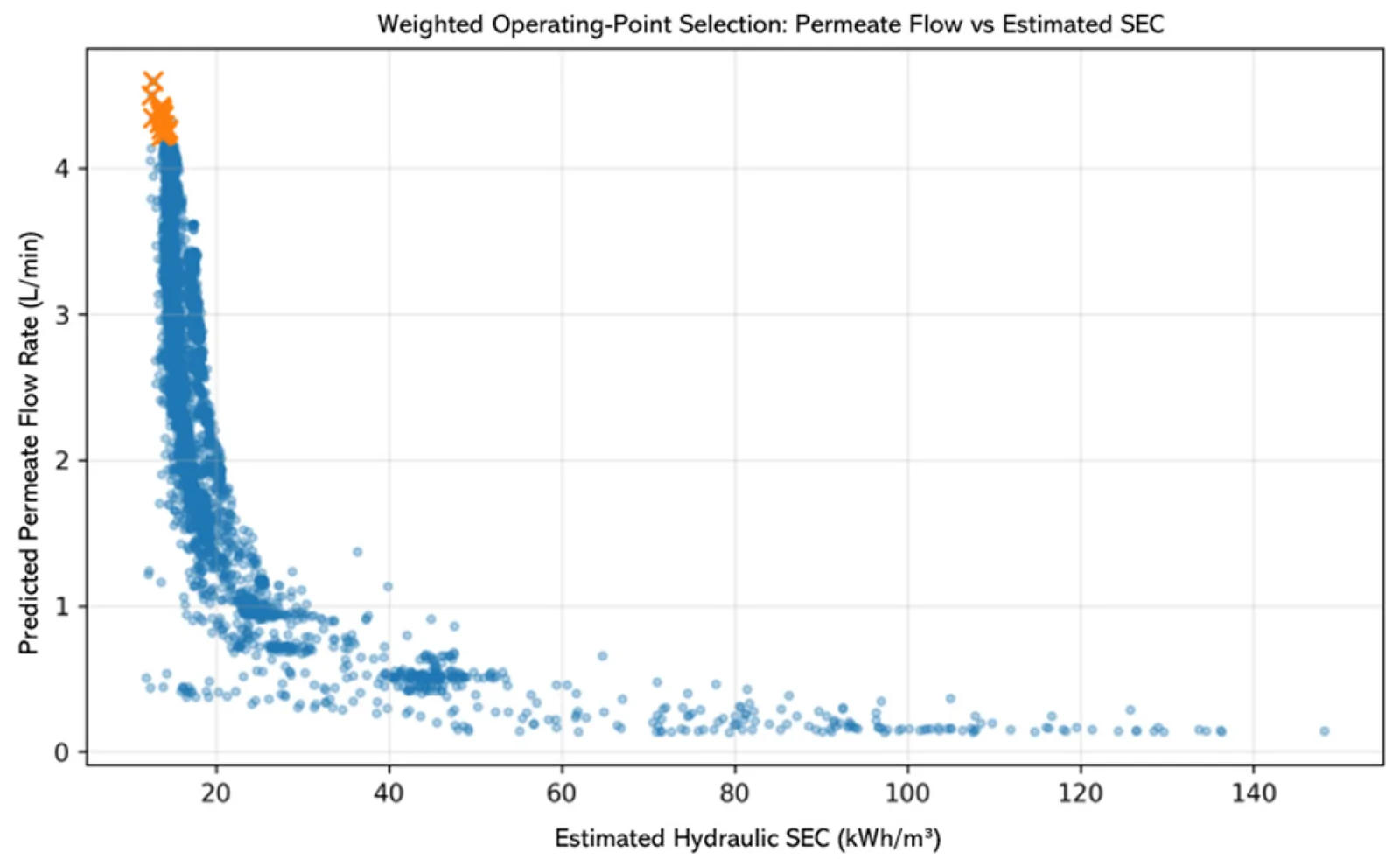
Weighted operating-point selection: predicted permeate flow versus estimated hydraulic SEC. Blue dots represent the candidate operating points, while orange crosses indicate the selected operating points.

As seen from Figure 11, the top-ranked candidate conditions are located mainly within the region of high water production and relatively low SEC. This indicates that the weighted ranking framework identifies candidate operating conditions representing a favorable permeate flow–SEC trade-off within the analyzed dataset. However, practical feasibility was not established because membrane-manufacturer pressure limits, scaling/fouling, recovery, and permeate-quality constraints were not applied.

**Figure 12:**
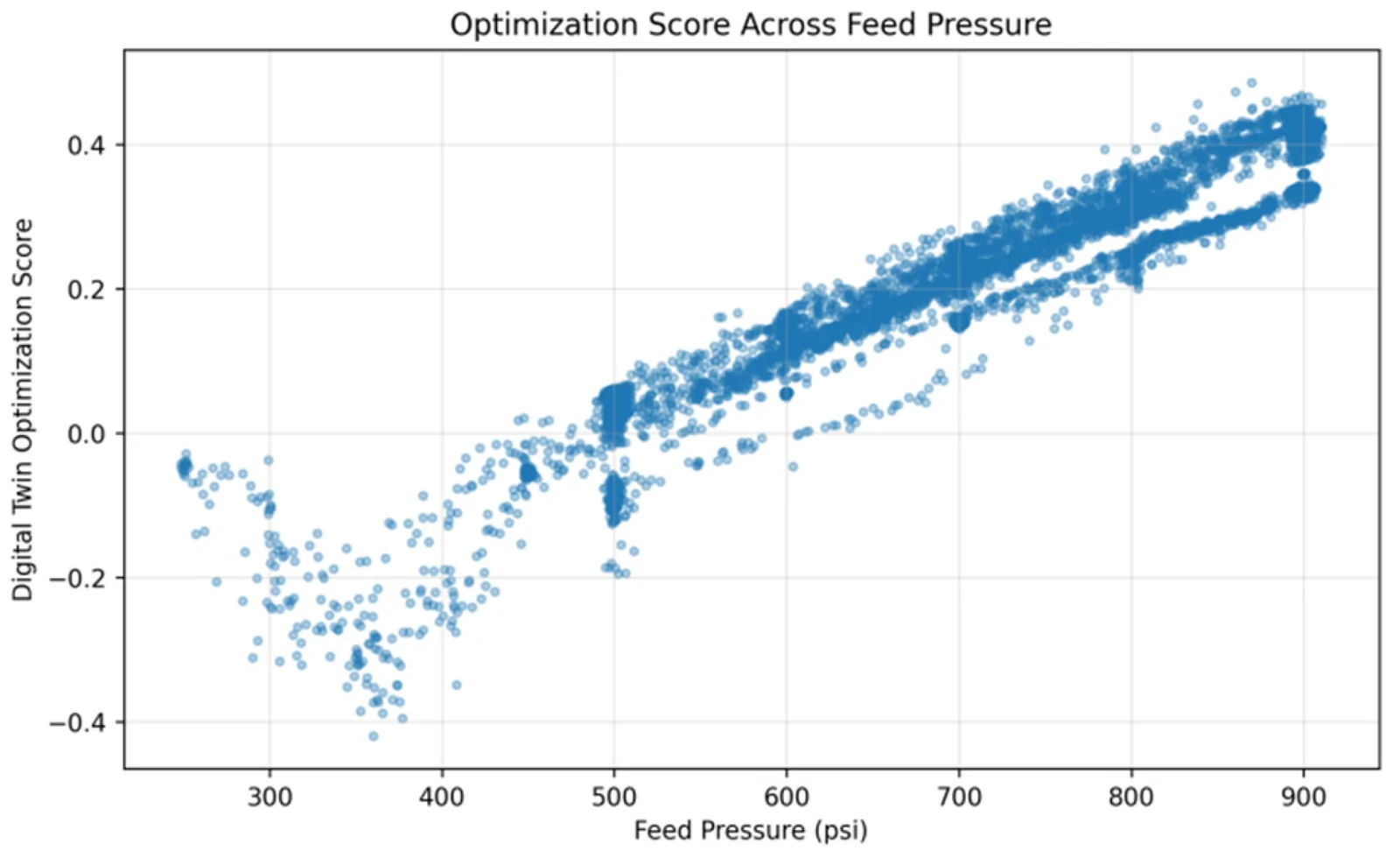
Weighted multi-criteria score across feed pressure.

Figure 12 shows that the ranking score generally increased with feed pressure within the observed data range; however, pressure alone did not determine the selected operating points. Higher pressure can increase freshwater production while also increasing pump-power demand. Therefore, favorable operating points were identified mainly from the freshwater–SEC trade-off, with conductivity treated as a secondary indicator. The observed pressure trend is therefore interpreted descriptively. The analysis does not claim that the ranking is independent of pressure or that the highest-pressure experimental runs are automatically feasible or optimal.

Accordingly, the weighted scoring framework supports RO operating-point screening under Kuwait solar-availability conditions; it does not represent a PV-constrained RO plant model. The proposed ranking approach considers multiple performance criteria simultaneously when screening candidate operating conditions, rather than relying on a single performance indicator.

It is extremely important for the system under study due to the great number of operating conditions that can be provided in different weather conditions.

### 5.6. Reliability of the Results and Limitations of the Current Dataset

The reliability of the freshwater model is justified for the range of the dataset as the test points are near the prediction line and the R2 value is high. The energy model is effective at optimizing trends but is still difficult for high-SEC outliers. The water quality model is underdeveloped and is a preliminary model. This variation in the outputs is anticipated because flow and energy are more closely related to pressure and flow rate, and conductivity is more closely related to other specific membrane and chemistry parameters. A principal limitation is that the NASA POWER and NREL datasets originate from independent measurement sources and were coupled computationally rather than collected from the same physical PV-RO plant. Therefore, the results demonstrate the methodological feasibility of the proposed framework but do not constitute field validation of a Kuwait PV-RO installation. Future validation should use co-located measurements of PV power, pump performance, feed-water properties, permeate quality, and operating setpoints from a pilot system in Kuwait. Another restriction is that the PV power values were not considered as a detailed PV array design model, but as a driver of availability. Module temperature, inverter efficiency, dust soiling, tilt optimization and battery/accumulator dynamics could be incorporated into a more advanced PV model. Because the models were trained on a specific experimental dataset, their predictive performance outside the range of observed operating conditions should be interpreted cautiously. Accordingly, the reported performance metrics are interpreted as point estimates within the observed data range; repeated grouped validation, confidence intervals, and uncertainty propagation into the ranking were not performed. Residual bias was not separately quantified in the current analysis. Because experiment type may encode waveform-class information, model performance is interpreted as conditional on the represented NREL experiment categories; no leave-one-experiment-type-out validation was performed. The present model comparison is limited to Gradient Boosting, Random Forest, and XGBoost; simple, linear, mechanistic, and direct-equation baselines were not evaluated. Additional residual, run-wise, SHAP, and uncertainty-band diagnostics were not available from the archived analysis and are reserved for future validation.

The main remaining methodological risk is that the study uses an open laboratory RO dataset rather than a dedicated Kuwait pilot PV-RO unit. Detailed membrane material, model, active area, module geometry, and manufacturer operating limits were unavailable in the processed source metadata; therefore, flux and membrane-compaction interpretations remain qualitative. Detailed feed-water chemistry, including pH, alkalinity, hardness, sulfate, silica, organic matter, and ionic composition, was also unavailable; therefore, scaling and fouling were not evaluated. Complete hyperparameter, software-version, and tuning records were not available for independent reconstruction; accordingly, claims of full computational reproducibility have been moderated.

## 6. Conclusions

In this work, we developed an offline digital-twin-assisted decision-support framework for dynamic RO under Kuwait solar-availability conditions. We have developed process-informed feature engineering and combined meteorological and solar data along with RO record to calculate recovery ratio, salt rejection efficiency, pumping power, specific energy consumption (SEC), pressure-rate characteristics, lag feature, and margin of PV power as operational indicators.

The developed machine-learning algorithms showed high accuracy in predicting freshwater production and Gradient Boosting provided better results compared to other approaches used in this paper. Energy prediction had satisfactory results, while permeate conductivity estimation still needs improvement. Accordingly, the current framework should not be used for definitive water quality control. Thus, in addition to the current set of parameters and their characteristics, some more physical data about the membranes’ condition and water chemistry could be needed.

The results demonstrate the potential of the proposed offline digital-twin-assisted decision-support framework for analyzing dynamic RO behavior and screening candidate operating conditions within the observed NREL dataset. The weighted scoring procedure identifies favorable candidate records under a Kuwait solar-availability context; however, these results should not be interpreted as field-validated PV-RO integration, experimentally demonstrated process improvements, or practically validated optimal operating conditions. The proposed framework provides a basis for future physically coupled PV-RO implementation using co-located field measurements.

Overall, the strongest contribution is not the use of machine learning alone, but the coupling of dynamic RO membrane behavior, process-informed indicators, and renewable energy availability into a transparent membrane-process decision framework.

Future work will focus on field-measured PV-RO data, detailed photovoltaic performance, membrane fouling, and water chemistry. From an environmental perspective, the framework may support lower-energy freshwater production and reduced dependence on fossil-based electricity, while future field studies should also evaluate brine management and life-cycle impacts.

## Figures and Tables

**Table 1:** Comparison of the proposed study with selected existing studies.

Study	Focus	Method Type	Main Contribution	Difference from Present Work
Freire-Gormaly and Bilton (2018) [33]	PV-powered RO intermittent operation	Experimental	Intermittent solar-powered RO behavior	No Kuwait solar coupling and no ML optimization
Leijon et al. (2020) [34]	Variable renewable power for RO	Review/case study	Renewable variability and desalination coupling	No data-driven digital-twin prediction layer
Brodersen et al. (2022) [36]	Wave-powered batch RO	Dynamic modeling	Direct-drive renewable RO concept	Focus on wave-energy design, not PV-RO ML
Sitterley et al. (2025) [39]	Dynamic feed pressure RO experiments	Experimental dataset/article	Ramping, sinusoidal and WEC-Sim RO data	Does not combine Kuwait solar-availability data and ML-based operating-point ranking.
Habieeb et al. (2023) [14]	AI for RO desalination	Comprehensive review	AI potential for predictive maintenance and optimization	Review only, no new Kuwait PV-RO dataset
Shahouni et al. (2024) [4]	AI for membrane behavior	Survey review	Membrane flux and fouling prediction methods	No PV-RO digital-twin application
Daaboub et al. (2024) [46]	Digital twins and ML for SWRO	Digital twin/ML study	Digital-twin concept for SWRO efficiency	Does not use the present dynamic NREL dataset and Kuwait solar case
Yogarathinam et al. (2025) [11]	ML under dynamic hydrodynamic RO	Supervised/deep ML	Dynamic feed flow and RO prediction	Focus is wave-powered RO, not Kuwait PV-RO energy margin

Present study: offline digital-twin-assisted decision-support framework for dynamic RO under Kuwait solar-availability conditions; hybrid data-driven framework; solar-availability data + dynamic RO data + ML prediction + weighted operating-point ranking; open-data coupling; water quality prediction remains limited by the available inputs.

**Table 2:** Dataset summary by experiment type.

Experiment Type	Samples	Mean Pressure (psi)	Mean Feed Flow (L/min)	Mean Permeate Flow (L/min)	Mean Flux (L m^−2^ h^−1^)	Mean SEC (kWh/m^3^)	Mean Recovery (%) *
membrane_integrity	35	693.75	NA	NA	NA	NA	9.465
ramp	169,838	774.89	22.008	2.941	23.846	17.445	12.930
rectified_sinusoidal	25	574.75	NA	NA	NA	NA	10.329
sinusoidal	27,815	612.48	18.939	2.061	16.641	21.520	9.821
steady_state	6040	699.96	20.285	1.955	15.848	25.248	9.097
wec_sim	36,398	717.02	20.194	2.560	20.758	15.928	12.518

NA: not available. * Recovery is a derived variable calculated according to Equation (1) using the available paired feed and permeate flow observations. Therefore, the reported mean recovery values should not be recalculated from the rounded category-level mean flow values presented in Table 2. NA indicates that the corresponding descriptive mean was unavailable.

**Table 3:** Descriptive statistics of RO operating and output variables.

Variable	Count	Mean	Std. Dev.	Min	25%	Median	75%	Max
feed_pressure_psi	240,151.00	745.39	147.57	247.85	625.99	794.21	894.93	911.95
feed_flow_lpm	240,041.00	21.334	2.943	8.954	19.097	21.784	24.038	26.600
feed_conductivity_ms_cm	240,041.00	54.806	2.479	51.102	53.323	53.572	55.616	61.911
feed_temperature_c	240,041.00	20.033	0.199	19.466	19.894	20.022	20.146	20.811
water_flux_lmh	240,041.00	22.343	8.978	0.000	15.223	23.550	31.573	45.331
permeate_flow_lpm	240,041.00	2.757	1.107	0.000	1.879	2.906	3.895	5.614
permeate_cond_us_cm	240,041.00	314.81	146.94	170.56	208.23	265.56	359.29	1163.94
reject_flow_lpm	240,041.00	18.146	1.777	10.106	16.764	18.413	19.866	20.595
estimated_pump_power_kw	240,041.00	2.681	0.826	0.392	1.931	2.837	3.528	3.946
sec_kwh_m3	237,777.00	17.847	8.214	2.363	15.078	15.896	17.956	199.75
recovery_percent_final	240,151.00	12.410	3.940	0.000	9.759	13.349	15.876	31.005
salt_rejection_percent_final	240,151.00	99.427	0.262	98.050	99.350	99.520	99.610	99.759

**Table 4:** Variable origin and potential mathematical target dependence.

Variable	Status	Mathematical Dependence
Feed pressure	Measured	Does not contain the prediction targets
Feed flow	Measured	Does not contain the prediction targets
Feed conductivity	Measured	Does not contain permeate flow or SEC
Feed temperature	Measured	Does not contain the prediction targets
Water flux	Derived	Contains permeate flow
Recovery	Derived	Contains permeate flow
Estimated pump power	Derived	Calculated from pressure and feed flow
SEC	Derived	Contains permeate flow
Permeate conductivity	Measured target	Used in apparent salt rejection
Salt rejection	Derived	Contains permeate conductivity
Pressure-rate/rolling/lag descriptors	Derived	Depends on their source variable
PV power margin	Derived	Scenario-level energy indicator

Note: Based on the model interpretation, the principal operational variables associated with permeate flow behavior were feed pressure, feed flow rate, and transient pressure descriptors; estimated pump power, feed pressure, and feed flow rate were directly related to SEC behavior; and feed conductivity and feed temperature were the most relevant available inputs for permeate conductivity prediction. Water flux, recovery, and SEC are mathematically dependent on permeate flow, whereas salt rejection is mathematically dependent on permeate conductivity; these dependencies are reported to prevent their interpretation as independent predictive information.

**Table 5:** Permeate-flow-rate prediction model performance.

Model	Target	MAE (L/min)	RMSE (L/min)	R^2^
Gradient Boosting	permeate_flow_lpm	0.106	0.161	0.981
Random Forest	permeate_flow_lpm	0.113	0.174	0.978
XGBoost	permeate_flow_lpm	0.131	0.185	0.976

**Table 6:** Specific energy consumption prediction model performance.

Model	Target	MAE (kWh/m^3^)	RMSE (kWh/m^3^)	R^2^	MAPE (%)
Random Forest	sec_kwh_m3	2.098	7.570	0.654	7.715
Gradient Boosting	sec_kwh_m3	2.495	7.575	0.654	9.086
XGBoost	sec_kwh_m3	2.915	8.058	0.609	11.332

**Table 7:** Permeate conductivity prediction model performance.

Model	Target	MAE (µS/cm)	RMSE (µS/cm)	R^2^	MAPE (%)
Random Forest	permeate_cond_us_cm	137.14	245.44	0.257	24.974
Gradient Boosting	permeate_cond_us_cm	123.86	245.50	0.256	20.378
XGBoost	permeate_cond_us_cm	125.75	248.77	0.237	20.511

**Table 8:** Full candidate set versus top-ranked candidate conditions.

Case	Permeate Flow (L/min)	SEC (kWh/m^3^)	Quality (µS/cm)	Pump Power (kW)	SEC Difference from Candidate Mean (%)	Permeate-Flow Difference from Candidate Mean (%)
Baseline/All Candidate Conditions	2.750	18.500	310.39	2.677	0.000	0.000
Top-Ranked Candidate Conditions	4.344	13.691	229.14	3.623	25.992	57.924

Note: Conductivity values are descriptive and should not be interpreted as validated water quality improvements.

## Data Availability

The NASA POWER solar and meteorological data and the NREL/DOE dynamic RO records are publicly available from the repositories cited in the References. No additional public code or processed-data archive is claimed in this study.
